# Machine Learning Logistic Regression Model for Early Decision Making in Referral of Children with Cervical Lymphadenopathy Suspected of Lymphoma

**DOI:** 10.3390/cancers15041178

**Published:** 2023-02-12

**Authors:** Eline A. M. Zijtregtop, Louise A. Winterswijk, Tammo P. A. Beishuizen, Christian M. Zwaan, Rutger A. J. Nievelstein, Friederike A. G. Meyer-Wentrup, Auke Beishuizen

**Affiliations:** 1 Department of Pediatric Hemato-Oncology, Princess Máxima Centre for Pediatric Oncology, Heidelberglaan 25, 3585 CS Utrecht, The Netherlands; 2 Department of Pediatric Hematology and Oncology, Erasmus Medical Centre-Sophia Children’s Hospital, Wytemaweg 80, 3015 CN Rotterdam, The Netherlands; 3 Division Imaging & Oncology, Department of Radiology & Nuclear Medicine, University Medical Centre Utrecht, Heidelberglaan 100, 3584 CX Utrecht, The Netherlands

**Keywords:** lymphoma, pediatric, children, cervical, lymphadenopathy, diagnostic model, non-Hodgkin lymphoma, Hodgkin lymphoma, diagnosis

## Abstract

**Simple Summary:**

Cervical lymphadenopathy is common in children. A decision model for detecting high-grade lymphoma in children with cervical lymphadenopathy is currently lacking. Most previous studies identified individual predicting factors for lymphoma, a few created multivariate models, but none of these were sufficiently discriminative for application in clinical practice. We have developed a 12-factor diagnostic scoring model with machine learning logistic regression that is highly sensitive and specific in detecting high-grade lymphomas. This diagnostic model facilitates early decision making in children with cervical lymphadenopathy suspected of lymphoma. Its application may enable early referral to a pediatric oncologist in patients with high-grade lymphoma and may reduce the number of referrals in patients with benign lymphadenopathy, thus preventing unnecessary invasive procedures, such as biopsies.

**Abstract:**

While cervical lymphadenopathy is common in children, a decision model for detecting high-grade lymphoma is lacking. Previously reported individual lymphoma-predicting factors and multivariate models were not sufficiently discriminative for clinical application. To develop a diagnostic scoring tool, we collected data from all children with cervical lymphadenopathy referred to our national pediatric oncology center within 30 months (*n* = 182). Thirty-nine putative lymphoma-predictive factors were investigated. The outcome groups were classical Hodgkin lymphoma (cHL), nodular lymphocyte-predominant Hodgkin lymphoma (NLPHL), non-Hodgkin lymphoma (NHL), other malignancies, and a benign group. We integrated the best univariate predicting factors into a multivariate, machine learning model. Logistic regression allocated each variable a weighing factor. The model was tested in a different patient cohort (*n* = 60). We report a 12-factor diagnostic model with a sensitivity of 95% (95% CI 89–98%) and a specificity of 88% (95% CI 77–94%) for detecting cHL and NHL. Our 12-factor diagnostic scoring model is highly sensitive and specific in detecting high-grade lymphomas in children with cervical lymphadenopathy. It may enable fast referral to a pediatric oncologist in patients with high-grade lymphoma and may reduce the number of referrals and unnecessary invasive procedures in children with benign lymphadenopathy.

## 1. Introduction

Lymphadenopathy is a common clinical finding in children and adolescents and can occur at any age [1,2,3,4,5,6,7]. In most cases it is caused by non-malignant conditions, such as infectious diseases, and therefore requires an empiric approach [8,9,10,11,12]. However, it can be a manifestation of malignancy, in particular lymphoma [13]. There are different types of lymphomas that occur in children. It is important that high grade lymphomas, such as classical Hodgkin lymphoma (cHL) and non-Hodgkin lymphoma (NHL), are diagnosed early. cHL occurs most common in adolescents, whereas the incidence of NHL increases steadily throughout childhood [14,15]. cHL presents most often with cervical lymphadenopathy, which can be slowly or rapidly progressive [16]. NHL represent a diverse group of lymphoid malignancies. Clinical presentations of NHL in children vary and depend on the histologic subtype, the extent of the disease, and the primary site of the tumor [14]. For example, T-cell lymphoblastic lymphoma (T-LBL) most commonly presents with peripheral lymphadenopathy with respiratory distress from mediastinal involvement, whereas anaplastic large cell lymphoma (ALCL) typically presents with painless lymphadenopathy with or without skin or subcutaneous involvement [14]. In both cHL and NHL, diagnostic delay may lead to the development of more widespread disease necessitating more intensive treatment, and it may increase treatment-related early and late toxicities [17,18,19,20,21]. On the contrary, nodular lymphocyte-predominant Hodgkin lymphoma (NLPHL) is a low-grade lymphoma. The clinical presentation mimics a benign cause of lymphadenopathy [22,23,24]. NLPHL is a subtype of cHL and accounts for approximately six percent of patients diagnosed with HL [25]. This lymphoma type has a cure rate of 100%, and therefore, some delay in diagnosis or referral is acceptable [22,25,26]. Due to these differences in diagnosis, pediatricians face a diagnostic dilemma: they should identify children in need of prompt referral to the pediatric oncologist, while avoiding unnecessary referral, and invasive evaluation, including biopsy, in most children.

Currently, the only existing guidelines on when to perform a biopsy are ambiguous and the current available literature is inconsistent about prediction factors for lymphoma in children with lymphadenopathy [27,28,29,30]. There are several factors associated with malignant lymphoma in univariate analysis, for example supraclavicular masses [31,32,33], lymph node size [32,34,35], mediastinal enlargement [32,33,35], elevated lactate dehydrogenase (LD) [8,32,35,36], and generalized disease [32,33]. There are only a few studies which were able to perform multivariate analysis, but none of these were sufficiently discriminative for application in clinical practice [31,34,37]. Some factors in univariate analysis are considered “red flags” and require immediate referral. However, not all patients present with the so called “red flags” and there is no literature about the value of the combination of prediction factors [27,28,29,30]. Moreover, integration of novel diagnostic biomarkers in the work-up for lymphadenopathy has also not been reported yet. TARC (CCL-17) is a novel diagnostic biomarker in children with cHL [38,39]. TARC is a chemokine that can be measured in plasma or serum by enzyme-linked immunosorbent assay (ELISA) [39]. It is not yet known if TARC can be used as a predictive factor in children with lymphadenopathy.

Machine learning algorithms are increasingly used in the medical field, with promising results [40,41,42,43]. The complex and unpredictable nature of human physiology has, in many circumstances, proven to be better described by machine learning algorithms. Machine learning makes it possible to uncover patterns, construct models, and make predictions by learning from training data [44]. It is particularly useful to uncover patterns that medical practitioners were unaware of or thought to be unlikely. Unlike the traditional predictive models that use selected variables for calculation, machine learning techniques can easily incorporate a large number of variables and newly available data to improve prediction performances [42,45].

Taken together, a diagnostic scoring model as a guideline for referral may be of great value in patients with clinical suspicion of lymphoma. Therefore, we have constructed a data-driven, diagnostic scoring model for the work-up of children with cervical lymphadenopathy suspicious of lymphoma.

Data-driven machine learning was highly suitable for this study as there were enough cases available. Additionally, machine learning models can quickly be retrained and used in practice. With this model, we aim to identify patients with high-grade lymphoma early. In the future, the implementation of a diagnostic scoring model may lead to less unnecessary referrals to the pediatric oncologist and less biopsies in patients with benign causes of lymphadenopathy.

## 2. Materials and Methods

### 2.1. Patient Inclusion

This is a retrospective, single center study, performed with patient data from the Princess Máxima Centre for Pediatric Oncology in Utrecht, the Netherlands, which is the only pediatric oncology facility in the Netherlands. We studied all patients (≤18 years) referred with lymphadenopathy suspected of lymphoma, either directly from general practitioners or from pediatricians, between June 2018 and December 2020 (*n* = 333). We included patients with cervical (including supraclavicular) lymphadenopathy or mass as the reason of referral (*n* = 182). We excluded patients who presented with lymphadenopathy or masses in other body regions, relapsed lymphoma, post-transplant lymphoproliferative disorders (PTLD), patients who had already started treatment for lymphoma elsewhere, and patients with genetic syndromes causing tumors.

A second cohort of patients was created consisting of all patients from January 2021 to January 2022 (*n* = 60). This cohort was used to test the reproducibility of the diagnostic model. Inclusion and exclusion criteria were the same as for the study group.

The study was approved by the Dutch Medical Research Ethical Committee Utrecht under trial number 21-073/C and number 16-739. All patients, and, when needed, parents or guardians, gave written informed consent.

### 2.2. Data Collection and Definitions

We collected data from electronic patient files. Two different researchers (E.A.M.Z. and L.A.W.) collected data independently. Uncertainties were discussed together and, if necessary, with the other researchers (A.B., R.J.N.) to reach a consensus. Due to the retrospective design, no standard formats were used for the description of the investigated variables. For example, involvement of the body regions was scored based on the radiology reports. Sometimes, one of the investigators was not certain how to interpret the radiology reports. This was discussed with the colleagues, and if necessary, the radiology investigations were re-analyzed by the involved radiologist of our study.

Data were processed anonymously and encrypted.

We identified predicting factors for lymphoma based on an extensive search of the literature using PubMed, Medline, and Embase. We searched for studies using Medical Subject Heading terms including “lymphadenopathy”, “child”, “adolescent”, and “lymphoma”. An overview of potential predicting factors based on this search of the literature and their results are given in Table S1 [8,31,32,33,34,35,36,37,38,46,47,48,49,50,51,52]. We identified 39 potential predictors and included these in our univariate analyses: age, gender, presence of B-symptoms, 11 laboratory parameters including TARC, and several imaging findings. These variables and their definitions are listed in Table S2.

The body regions of the involved areas were scored individually. An overview of the separately scored anatomical body regions and an explanation is provided in Table S3.

We used pathology reports primarily for defining the diagnosis; 158 out of 182 patients underwent biopsy, including all cases of lymphoma. Twenty-four patients were diagnosed without a biopsy, but based on clinical, radiological, microbiological, and laboratory results (twenty infectious/reactive lymphadenopathy, one venous malformation, one lymphangioma, one branchiogenic cyst, and one dermoid cyst).

We categorized the patients into 12 groups according to their diagnosis. The malignant diagnoses in the study population included: cHL, NLPHL, ALCL, primary mediastinal large B-cell lymphoma (PMBCL), diffuse large B-cell lymphoma (DLBCL), Burkitt lymphoma (BL), T-LBL, B-cell lymphoblastic lymphoma (B-LBL), and other malignancies (Langerhans cell histiocytosis (LCH)). Furthermore, there were three groups with benign causes of lymphadenopathy: reactive or infectious lymphadenopathy, progressive transformation of germinal centers (PTGC), and other non-malignant causes.

For the identification of predictive factors, we divided the outcome into the benign group and the malignant group for univariate analysis. However, the malignant group contained nine different diagnoses, which differ significantly in incidence and clinical presentation. Therefore, we subdivided the group into five categories for multivariate analysis: cHL, NLPHL, NHL, other malignancies, and the benign group. In brief, we collected data from electronic patient files. We identified 39 potential predictors based on an extensive search of the literature. We used pathology reports for defining the diagnosis.

### 2.3. Statistical Analysis

For univariate analysis, the Fisher’s exact test was used for binary variables to determine differences between the malignant and benign group. Laboratory values were analyzed both as continuous factors as well as binary variables with a cut-off point to investigate in which way they predict best for lymphoma. To develop a cut-off point, we developed a receiver operating characteristic (ROC) curve to determine the best cut-off point. We calculated the odds ratios (OR) with 95% confidence intervals (CI) to investigate the best predicting factors. In the case that one of the predictors was not present in either the benign or malignant group (*n* = 0), the OR was set as 0. In that case, we used the *p*-value for the selection for multivariate analysis. A *p*-value of <0.01 was considered statistically significant.

Since TARC is a new biomarker and only known as a marker for cHL so far, TARC was also evaluated as variable between cHL, NLPHL, NHL, and the benign group. We used SPSS Statistical software (version 27.0, IBM, Armonk, NY, USA) for univariate analysis.

NLPHL is a unique form of lymphoma. Its clinical presentation mimics a benign cause of lymphadenopathy [22,23,24]. Therefore, we investigated whether there are predicting factors to distinguish between NLPHL and the benign group.

We performed multivariate data analysis using Python Programming software (version 3.8.8). We decided on a data-driven approach instead of using the more traditional bio statistical methodologies. We chose this novel approach for efficiency and automation in finding the best model, as well as the removal of human bias in decisions. We used the most relevant variables based on univariate analysis and only included variables which can be used in general clinical practice. Using univariate analysis for feature selection does not address multicollinearity, whereas multivariate techniques, such as LASSO, are more successful in addressing this [53]. To prevent collinearity from biasing our results, we tested the correlation of our input predictors against each other with a Kendall Tau test. Besides that, we also performed further feature selection using the multivariate technique least absolute shrinkage and selection operator (LASSO) and extracted the feature importance in LASSO to use in the assessment.

We built a machine learning model using logistic regression in the scikit-learn package version 0.24.1.

We tested out several different model types, including easily explainable versions, such as decision trees and logistic regression, and the higher quality models, such as random forests and support vector machines [54,55]. Details of the different model types and the comparisons of the different models are provided in the Text S1.

Logistic regression was chosen for its relatedness to other human-made models and comprehensibility and its limited compromise on model quality. The machine learning model is binary (positive or negative) and assumes that no missing variables occur. However, there were some missing variables. We decided to mark all missing variables as negative, since we hypothesized a diagnostic test is not performed when the suspicion of abnormalities is low.

The model calculated the importance of a variable and gave it a weighing factor using logistic regression. A more detailed explanation of the weighing factor and the formula is provided in the Text S2. In model training, we set false negatives to be twice as bad as false positives, as, in general, false negatives are to be avoided when dealing with potential malignant disease. To validate the model quality, we used a 5-fold cross validation on the complete dataset for quality calculation.

A 5-fold cross-validation (CV) was applied to test the model quality on the dataset directly without overfitting and by using the entire set in training. In the 5-fold CV, the data are first randomly split into five parts. Then, the model was trained on four parts and we validated the quality with the fifth part. We then applied the same technique five times, for each combination once. The results of the 5-fold CV are the averaged values of the quality metrics obtained from the five tests. We evaluated the model in terms of sensitivity, specificity, and likelihood:

$$Sensitivity=\frac{True\;Positives}{True\;Positives+False\;Negatives}$$

$$Specificity=\frac{True\;Negatives}{True\;Negatives+False\;Positives}$$

$$Likelihood\;Ratio+=\frac{Sensitivity}{1-Specificity}$$

$$Likelihood\;Ratio-=\frac{1-Sensitivity}{Specificity}$$

Besides these quality metrics, we also visualized the receiver operator curve (ROC) and calculated the area under the ROC curve, also known as the area under the curve (AUC). To define the best cut-off in the ROC curves, we used the Youden Index in combination with expert opinions.

$$Youden^{\prime }s\;Index=Sensitivity+Specificity-1$$

Moreover, we also used the patient dataset to identify potential overfitting.

After finishing the multivariate model, we tested the model in a second (test) cohort. We first investigated whether the test group and the study group are similar enough, such that we can use the test set to test the model. We calculated whether the input variable distribution was significantly different between the study and test cohort, for the whole set combined, and for the malignant and benign cases separately. Afterwards, we tested the model for its quality and lack of overfitting in the second (test) cohort.

## 3. Results

### 3.1. Study Population and Baseline Characteristics

**Figure 1:**
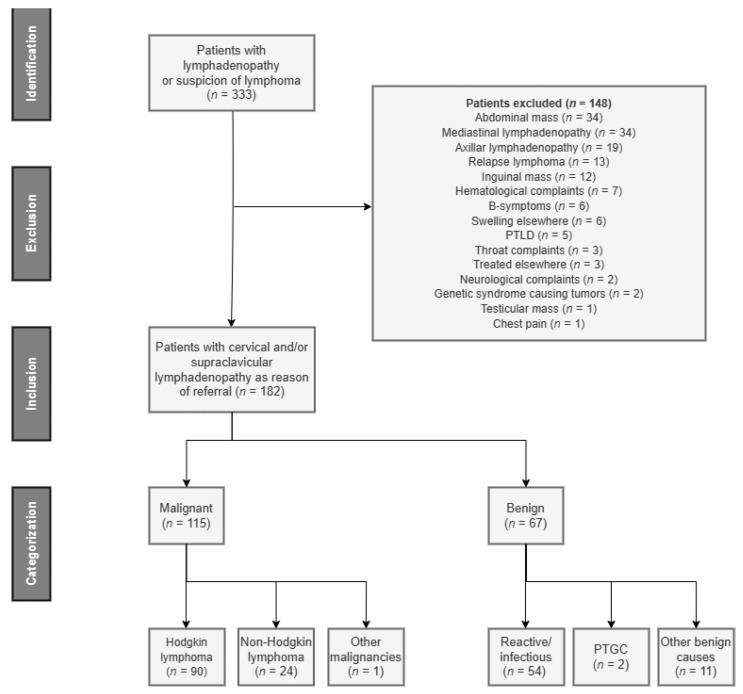
Flow diagram of study participants. In total, 333 patients were referred with lymphadenopathy. Of these, 151 patients were excluded based on our exclusion criteria. There were 182 patients referred with cervical/supraclavicular lymphadenopathy. Of these 182 patients, 115 patients were diagnosed with lymphoma and 67 with benign causes of lymphadenopathy. Abbreviations: PTLD—post-transplant lymphoproliferative disorders; PTGC—progressive transformation of germinal centers.

In total, 182 patients were referred with cervical lymphadenopathy and included in the study (consort diagram, Figure 1). The malignant group contained 115 patients, including 79 cHL, 11 NLPHL, 24 NHL, and 1 LCH patient. The benign group contained 67 patients, including 48 with reactive/infectious lymphadenopathy (Table 1). Baseline characteristics are provided in Table 1. The test set contained 60 patients: 40 patients with malignancies and 20 with benign lymphadenopathy. The patient characteristics of the test group were not statistically different from the study group (Table 1).

### 3.2. Univariate Analysis Identified 29 Predictive Factors for Lymphoma

Univariate analysis of all variables identified the significant variables (*p* < 0.01) as significant predictors for high-grade lymphoma. The results of the univariate analysis are presented in Table 2. Odds Ratios and a 95% CI showed the predicting value of the different variables. We found that *p*-values for the laboratory values as continuous variables were comparable with the *p*-values when analyzed as dichotomous variables. Odds ratios were easier to interpret when analyzed as dichotomous variables due to the high range of some of the laboratory variables. Moreover, a cut-off point is very useful in clinical practice. Therefore, we decided to use a cut-off point for laboratory values for further analysis.

As described in the statistical analysis, we performed some extra analysis on TARC to investigate whether it is a predictive factor for all types of lymphoma. TARC was found to be a predictive factor for high-grade lymphoma in our univariate analysis. However, this was only due to the 79 cases of cHL in the malignant group. TARC values were significantly higher in the cHL group compared to the NLPHL, NHL, and the benign group (median 7.207 pg/mL in cHL versus median 66 pg/mL in NLPHL, median 155 pg/mL in NHL and median 88 pg/mL in the benign group, *p* < 0.001). The ROC analysis showed that TARC is an excellent predictor for cHL (AUC 0.954, 95% CI 0.920–0.989), but not for other types of lymphoma (AUC 0.567, 95% CI 0.429–0.704).

Based on the results of the univariate analysis, we decided to include all variables with a *p*-value of <0.001 combined with an OR of larger than five and a CI of greater than one in the multivariate analysis. The presence of trachea deviation, obstructed airway, and vena cava superior syndrome were very specific for lymphoma. However, we excluded these factors from the multivariate analysis, since these were almost only present in patients with enlarged mediastinum, and we decided to add enlarged mediastinum in our multivariate analysis, since this can be established on a routine X-thorax. The same accounted for the presence of cervical level IV lymph nodes and the presence of supraclavicular involvement in general, since level IV refers to a supraclavicular region. We, therefore, included only supraclavicular involvement.

Univariate analysis of the NLPHL and the benign group showed that patients with NLPHL were significantly more often male (85% versus 50% *p* = 0.02), had more pathological lymph nodes visible on ultrasound (92% versus 36% *p* < 0.001), and never had B-symptoms (0% versus 30% *p* = 0.02). The C-reactive protein (CRP) and erythrocyte sedimentation rate (ESR) were significantly lower in patients with NLPHL.

### 3.3. Multivariate Analysis Creates a Diagnostic Model for Predicting Lymphoma

For multivariate analysis, outcomes were split into five diagnostic groups. The group with other malignancies contained only one patient with LCH and this patient was therefore excluded. We decided to exclude NLPHL patients for the creation of the model, because NLPHL showed no predictive quality in univariate analysis and because of the lack of medical need for immediate care for these patients. However, we did include NLPHL in the visualization of the outcome of the scoring model to show the differences between the groups. Therefore, the model was created based on 171 patients.

In our first model, NHL was difficult to differentiate from cHL. According to the literature, lactate dehydrogenase (LD) is a well-known predictive factor for NHL, but not for HL [8,56,57]. LD was not statistically significant in our univariate analysis. We hypothesized that this was caused by the outcome comparison grouped in malignant and benign, since the malignant group contained mostly HL cases. For that reason, we tested LD in our multivariate model as well. Adding LD gave a better predictive power for NHL, and therefore LD was included in the final model.

We started the multivariate analysis with 13 variables, based on the univariate analysis. One variable gave no added value in the multivariate analysis (CRP), and was therefore rejected. The Kendall Tau test showed that most predictors correlate with other predictors, and therefore raise concern for collinearity. LASSO, on the other hand, attributed a positive feature importance to all 12 variables; although, the contribution of some of the variables was relatively low. Since all variables contributed positively, we decided to keep these 12 variables in the final model.

**Figure 2:**
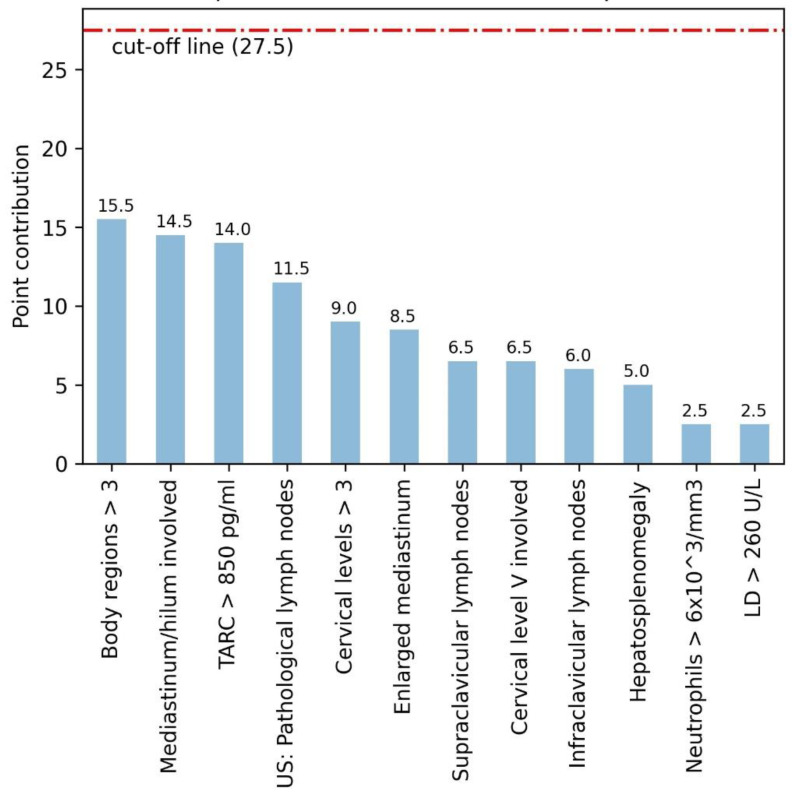
Factors included in the final multivariate scoring model with their weighing factors. The blue columns represent the weighing factors of the variables. The red line represents the cut-off for referral. Abbreviations: TARC—thymus and activation regulated chemokine; LD—lactate dehydrogenase; US—ultrasound; hepatosplenomegaly—hepatomegaly, splenomegaly, or hepatosplenomegaly.

The following variables were included in the final scoring model (listed based on weighting factor): involvement of more than three body regions; involvement of mediastinum or hilum; TARC > 850 pg/mL; the presence of pathological lymph nodes on ultrasound; involvement of more than three cervical levels; enlarged mediastinum; cervical level V involvement; involvement of supraclavicular lymph nodes; involvement of infraclavicular lymph nodes; enlarged liver and/or spleen; neutrophils > 6.0 × 10^9^/L and LD > 260 U/L. The variables and their corresponding weighing factors are distributed in Figure 2 (maximum score 102).

**Figure 3:**
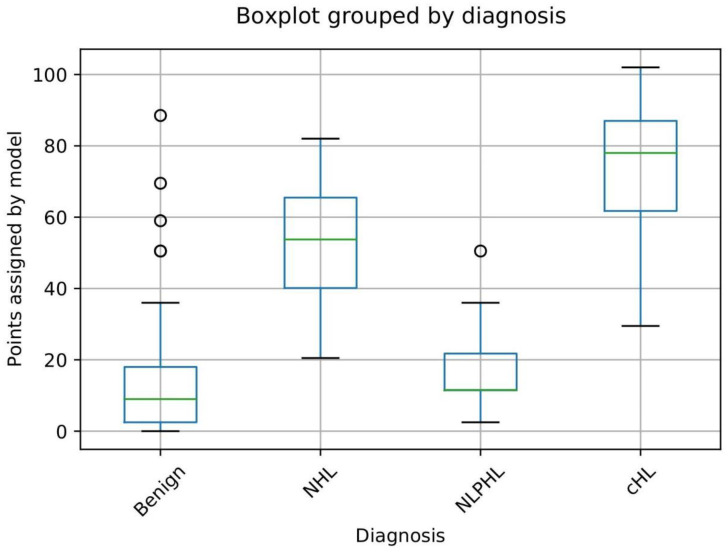
Overview of scored points according to our diagnostic model distributed in four outcome groups. The diagnostic model could discriminate excellently (*p* < 0.001) between the benign group (median 9.0, 25–75 percentiles 2.5–18.0 points) and the cHL group (median 78.0, 25–75 percentiles 62.0–87.0 points) and between the benign group and NHL group (median 54.0, 25–75 percentiles 40.0–65.5). The model could not discriminate between NLPHL and the benign group. Abbreviations: NHL—non-Hodgkin lymphoma; NLPHL—nodular lymphocyte-predominant Hodgkin lymphoma; cHL—classical Hodgkin lymphoma.

The model discriminates excellently between the benign group and cHL and NHL patients (Figure 3). CHL patients scored 78 points as median value (25–75 percentiles 62.0–87.0) versus 9.0 points (25–75 percentiles 2.5–18.0) scored in the benign group, *p* < 0.001. NHL patients scored 54.0 points (25–75 percentiles 40.0–65.5) versus 9.0 points (25–75 percentiles 2.5–18.0) in the benign group, *p* < 0.001. As expected, the model was not able to discriminate between NLPHL and the benign group (*p* = 0.23) (Figure 3).

**Figure 4:**
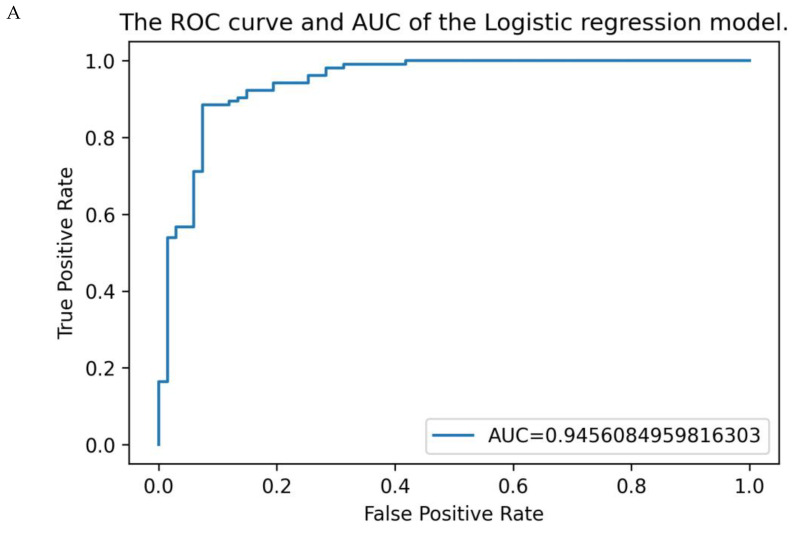

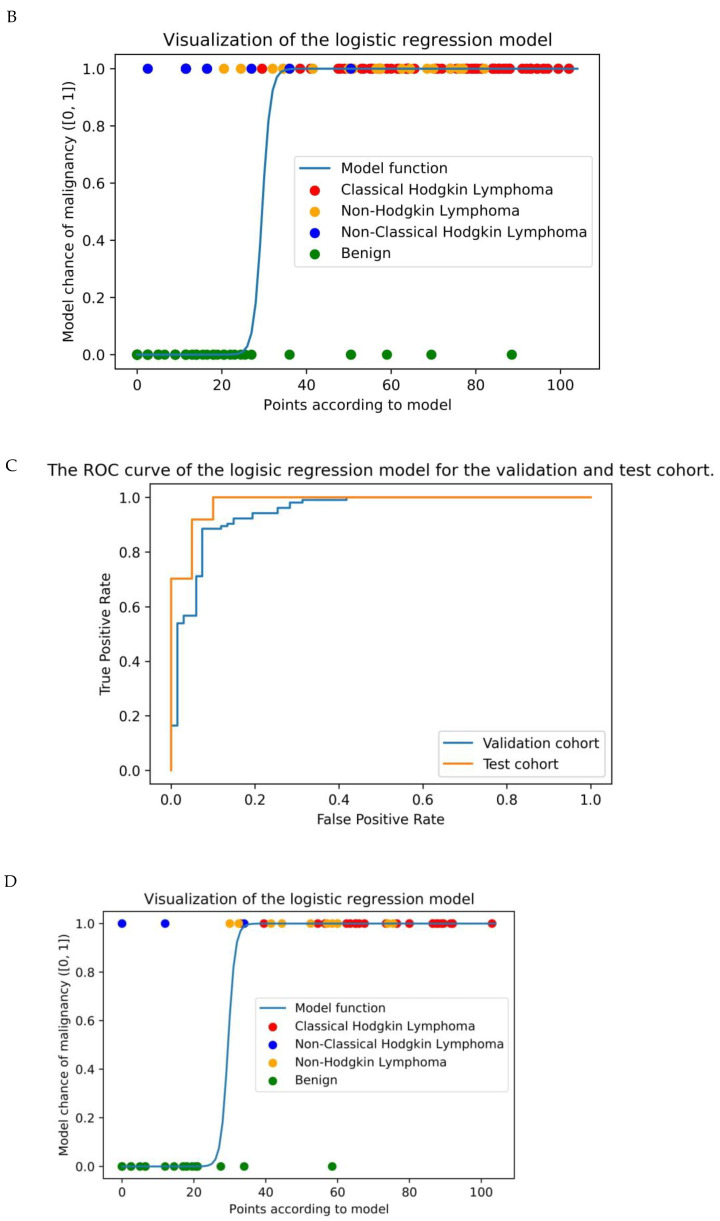
ROC curve and logistic regression curve scored by the diagnostic model in the study population (A) (*n* = 171) and the test group (B) (*n* = 57). (**A**) ROC curve of the study group, with an AUC of 92% (95% CI 87–96%); (**B**) the blue line represents the model function. With a cut-off point of 27.5, the figure demonstrates that all cHL and 22 out of 24 NHL were detected with this diagnostic model and 61 out of 67 patients with a benign cause were diagnosed as benign according to the model; (**C**) ROC curve of the study group and test group together. The test group had a comparable AUC of 98% (95% CI 95–100%); (**D**) with a cut-off point at 27.5, the figure demonstrates that 55 out of 57 patients were diagnosed correctly. All 37 patients with cHL and NHL were diagnosed correctly, and 18 out 20 patients with a benign cause were diagnosed as benign according to the model.

After CV, the final model provided a sensitivity of 95% (95% CI 89–98%) and a specificity of 88% (95%CI 77–94%). The ROC curve of the logistic regression model is shown in Figure 4A. The AUC was 92% (95% CI 87–96%). The logistic regression model curve is shown in Figure 4B. The ROC curves of the outcomes of the cross validation are shown in Figure S1. The sensitivity and specificity were similar to the alternative machine learning models, confirming our choice of using the explainable logistic regression (Table S4). ROC curves of the different models are shown in Figure S2. When keeping the numeric values continuous when training the model, we observed a similar sensitivity of 94% ((95% CI 87–98%), but a lower specificity of 76% ((95% CI 64–85%), potentially due to overfitting on the continuous scales. Youden’s Index was the highest at 86% with a score of 33.5. However, this score does not take into account that false negatives are more costly than false positives; therefore, we chose to reduce the score to 27.5. With this cut-off point, there were two false negative cases of NHL, and six false positive cases in a total of 171 patients.

After creating the model, we tested it on the second (test) cohort. First, we compared the test group and the study group for overall differences. For all included variables, there were no significant differences between the test group and study group, in total; between the two malignant groups; and between the two benign groups (*p* < 0.01). In the test cohort, the model provided a sensitivity of 100% (95% CI 80–100%) and a specificity of 93% (95% CI 80–98%) for detecting high-grade lymphoma. The ROC curve of the study and test cohort together is shown in Figure 4C. The AUC was 98% (95% CI 95–100%). The logistic regression model curve is shown in Figure 4D.

The final model follows the following formula:

$$R=\sum a_{f}x_{f}$$

With *f* being any of the 12 predictors, *x* being the presence of feature *f* (1 if condition applies, 0 if not), and *a* being the weighing factor (Figure 2). R corresponds to the resulting score that needs to be above 27.5 to result in a classification to have a very high chance to be malignant.

#### Added Value of the Model Compared to the Literature

An enlarged mediastinum, pathological lymph nodes, supraclavicular involvement, and extended disease are sometimes described in the literature as “red flags”. In the univariate analysis, 64 out of 66 patients (97%) with an enlarged mediastinum were diagnosed with a malignancy. The multivariate model recognized all these patients as malignant and could not identify the two patients without malignancy as benign. For the other red flags, the positive predictive value of the variables was lower; 91% for more than three body regions involved (extended disease), 84% for supraclavicular involvement, and 81% for pathological lymph nodes on ultrasound. The model showed added value for these patients; 2 out of 8 (25%) patients with more than three body regions were identified correctly as not malignant with the model, 13 out of 19 (68%) patients with supraclavicular involvement were identified correctly, and 18 out of 24 (75%) with pathological lymph nodes were identified correctly. The six patients that the model misdiagnosed had both supraclavicular involvement and pathological lymph nodes, and more than three body regions involved.

One of the two false negative patients had none of the red flags. For this patient, adding red flags for immediate referral would not have made a difference. The other patient did have more than three body regions involved, and for this patient, immediate referral would have made the difference.

### 3.4. Added Value of TARC

TARC is a novel biomarker that is so far not routinely used in the work-up for pediatric lymphadenopathy. Therefore, we analyzed the additional value of TARC in our model. To investigate this, we set all TARC values to be above the cut-off of 850 pg/mL, since we hypothesized missing a malignant case would be more harmful than an unnecessary referral. In that case, another 21 out of the 67 (31%) benign patients would have been referred unnecessary. If we set all TARC values to be normal (<850 pg/mL), one patient with malignancy would be missed. So, TARC has major value to the model, especially for avoiding unnecessary referral.

## 4. Discussion

We developed a machine learning diagnostic model with 12 variables, including the novel biomarker TARC, which can serve as an important decision tool for the adequate referral of children with cervical lymphadenopathy. Lymphadenopathy is a frequent problem in children, and therefore it deserves a targeted approach. Timely recognition of high-grade lymphomas is a prerequisite. On the other hand, unnecessary referral to a pediatric oncologist leads to high stress in children and parents. Most studies identified individual predicting factors for lymphoma. A few studies created a multivariate model, but none of these were sufficiently discriminative for application in clinical practice [31,34,37]. Decision models could contribute to a targeted approach and have not yet been described in the literature. The high statistical power of this diagnostic model is very promising.

The model is intended as a diagnostic tool for patients with a high suspicion of malignant lymphoma. Based on a high sensitivity and specificity for diagnosing high-grade lymphoma in our cohort, this model appears to be very suitable for targeted decision making in children with cervical lymphadenopathy suspected of lymphoma. Patients with high-grade lymphoma will be identified and referred early. It may also prevent referrals to the pediatric oncologist in patients with a benign cause of lymphadenopathy, leading to less unnecessary biopsies as well. In our study, all biopsies in benign cases were performed to rule out malignancy. In retrospect, in almost all cases the application of our diagnostic tool would have prevented the referral to the pediatric oncologist and the biopsy that was performed. Therefore, our decision model will contribute to a reduction of unnecessary referrals and biopsies. Additionally, this could be highly beneficial for children and parents because of the stress that unnecessary referrals to a pediatric oncologist may cause. Finally, a more targeted approach will most likely lead to a higher cost-effectiveness of care.

In the Princess Máxima Centre for pediatric oncology, the diagnostic model is implemented during decision making about a biopsy in patients with cervical lymphadenopathy. Furthermore, the score is used for decision making when pediatricians in the Netherlands are consulting the Princess Máxima Centre about a patient with cervical lymphadenopathy, especially in the case of there being a long distance between the different hospitals. Before using the diagnostic model in general pediatric care, the model should be validated first in that setting. However, implementation will be easy, since all variables can be performed in general pediatric care. The diagnostic process for patients in a general pediatric practice often includes a broader work-up, including screening for infectious diseases, which is not taken into account in this study. To use the model, the following diagnostic investigations should be obtained: full physical examination, including all lymph node stations; an ultrasound of the neck region, focusing on the presence of pathological lymph nodes and the involved levels; an X-thorax to detect mediastinal enlargement, hilar and mediastinal lymphadenopathy, or lung lesions; an ultrasound of the abdomen, focusing on the presence of pathological lymph nodes, involvement of organs, and hepatosplenomegaly; TARC; LD; and neutrophil count. The investigations do not have to be performed at once; if a patient does not score points at the first investigation, the pediatrician can calculate if it is necessary to complete further investigations, based on the scoring points of the model. TARC may not be determined in every hospital, but could be sent to a neighboring hospital for determination. This work-up may be broader than the current pediatric approach. However, all proposed diagnostic tests are not invasive or are minimally invasive, and this diagnostic model will lead to less unnecessary referrals and invasive biopsies.

All variables used in our diagnostic model, besides TARC, have been described previously as separate predicting factors for lymphoma [8,32,33,37,46,47,48,49,50,51,56,57,58,59,60,61]. Our study confirmed that enlarged mediastinum is an absolute “red flag”, calling for prompt referral to the pediatric oncologist. The model referred all patients with enlarged mediastinum. For other known “red flags”, our model showed additional value in discriminating between benign and malignant disease in patients with these variables.

We have previously shown that TARC is a diagnostic marker for pediatric cHL [38,39]. The current study showed that TARC is a valuable diagnostic marker in patients with cervical lymphadenopathy as well. TARC has the greatest added value in patients with a benign cause of lymphadenopathy and in patients with cHL.

Despite the high sensitivity and specificity, the model resulted in two false negative and six false positive cases. One patient with B-LBL presented with a skin lesion and cervical lymphadenopathy without other signs of disease and was, therefore, missed as lymphoma. The other patient had BL; in this case, there was no ultrasound of the neck performed, and therefore we could not score several variables. This most likely has contributed to the false negative result. The six false positive cases were patients with extended lymphadenopathy. One patient was diagnosed with tuberculosis, one with atypical mycobacterial infection caused by an inborn IFNGR1 mutation, two with rheumatological disorders, and two with other infectious diseases. Four of these are rare disorders, sometimes mimicking lymphoma, thus justifying referral to the oncologist.

The diagnostic model is not suitable for the detection of NLPHL. NLPHL only accounts for 5–8% of pediatric HL patients [62] and most often presents with stage I or II disease [63,64]. All patients in our study achieved full remission after surgery alone or after three cycles of chemotherapy. The lymphadenopathy in these patients existed for a longer period (mean 8.9 months, range 1.5–42 months). We argue that it is acceptable that the model misses these NLPHL patients at the first assessment, since NLPHL is a low-grade tumor with a survival rate of 100% (22, 26). However, it is important that NLPHL will be detected, referred, and treated. We therefore recommend performing a lymph node biopsy if the cervical lymphadenopathy persists for more than 4 months after the first admission and cannot be explained otherwise.

A strength of this study is the data-driven approach instead of the traditional biostatistical, hypothesis-driven approach. The data-driven approach is highly efficient in creating the most powerful model and reduces human bias in decision making. The best predicting model was created by machine learning, based on the available data. Using machine learning also has its limitations. This data-driven approach might surface non-existing correlations by chance, due to bias being present in the data. Alternatively, the final model might be too difficult to understand by the ones supposed to use it. This may reduce adaption significantly. To address these concerns, the model was judged and evaluated by the investigators by comparing it with the existing literature. The most important limitation of this multivariate model is the potential collinearity of the variables. From a clinical perspective, collinearity is to be expected, since patients most often present with a combination of symptoms. Our correlation matrix raised suspicion for collinearity; however, LASSO showed a positive contribution for each of the variables, and therefore we decided to leave all variables in. Future research should reveal if the model can be adjusted or minimalized. Potential solutions to address collinearity in future research are the use of an algorithm, such as optimized Elasticnet. Another limitation of this study is the retrospective design. Data were collected from the electronic patient files. However, no standard formats for description of the variables existed, for example, the involvement of different body regions. For these variables, it would be of added value to describe this in standard formats and analyze prospectively. Moreover, due to the retrospective design, some missing values in different variables were found. The machine learning model in multivariate analysis was binary (positive or negative) and assumed that no missing variables occur. Therefore, we decided to mark all missing variables as negative, since we hypothesized a diagnostic test is not performed when the suspicion of abnormalities is low. This was a risk, especially if there was a higher percentage of missing values in the malignant group. This was the case in the presence of pathological lymph nodes on ultrasound. We expect that these variables would be given a higher weighing factor if the values were not missing. All of the abovementioned limitations highlight the need to retrain the model with prospective data in the future.

The authors acknowledge that this work is based on a patient population referred to the pediatric oncologist, making the diagnosis of malignancy more likely. The strength of this study population is the high number of lymphoma patients, which is not reported in the literature yet. Moreover, due to the centralization of pediatric oncology care in the Netherlands, this dataset contains all cases of lymphadenopathy referred to the pediatric oncologist within the whole country. On the other hand, this study is limited by the fact that the study patients are a selection of lymphadenopathy patients with suspicion of malignancy seen by general practitioners and referral hospitals, which is illustrated by the fact that only a few patients had single-node disease. Therefore, additional research is needed to explore the effectiveness of the model in general pediatric care. Furthermore, the feasibility of the model, which integrates different diagnostics, including the novel biomarker TARC, should also be tested in general pediatric care.

## 5. Conclusions

In conclusion, lymphadenopathy is a frequent problem in children, and therefore deserves a targeted diagnostic approach. Timely detection of high-grade lymphomas is a prerequisite. On the other hand, unnecessary referral to a pediatric oncologist leads to high stress in children and parents. Decision models could contribute to this targeted approach. Our 12-factor logistic regression machine learning diagnostic model is a crucial step forward towards a targeted diagnostic approach in children with cervical lymphadenopathy suspected of lymphoma. Our study results suggest that this model will support the identification of patients with high-grade lymphoma. This may reduce unnecessary referral to the pediatric oncologist and unnecessary biopsies. Future studies should focus on testing and validating our decision model for children with cervical lymphadenopathy in different clinical settings.

## Figures and Tables

**Table 1:** Baseline characteristics.

**Characteristics Malignant Group**	**Study Group,** **Malignant** ***n* = 115**	**Test Group,** **Malignant** ***n* = 40**	**Differences between** **Malignant Groups,** ***p*-Value**	**Differences** **between Test Group and Study Group in Total** ***p*-Values**
Gender	-	-	0.72	0.88
Male (%)	67 (58.2)	22 (55.0)	-	-
Female (%)	48 (41.7)	18 (45.0)	-	-
Age median (range)	14.0 (1–18)	14.2 (2–18)	0.53	0.80
Diagnoses (%)	-	-	0.26	-
Hodgkin lymphoma	90 (78.3)	29 (72.5)	-	-
cHL	79	26	-	-
NLPHL	11	3	-	-
Non-Hodgkin lymphoma	24 (20.9)	11 (27.5)	-	-
ALCL	5	1	-	-
PMBCL	1	0	-	-
DLBCL	3	1	-	-
BL	5	0	-	-
T-LBL	8	9	-	-
B-LBL	2	0	-	-
Other malignancy	1 (0.9)	0 (0.0)	-	-
Histiocytosis	1	0	-	-
Isolated lymphadenopathy (%) *	8 (6.9)	3 (7.5)	0.91	1.00
cHL	-	1	-	-
NLPHL	8	2	-	-
**Characteristics Benign Group**	**Study Group,** **Benign** ***n* = 67**	**Test Group,** **Benign** ***n* = 20**	**Differences between** **Benign Groups,** ***p*-Value**	**Not Applicable**
Gender	-		0.95	-
Male (%)	33 (49.3)	10 (50.0)	-	-
Female (%)	34 (50.7)	10 (50.0)	-	-
Age median (range)	12.5 (0–17)	13.5 (1–18)	0.51	-
Diagnoses (%)	-		0.19	-
Reactive/infective lymphadenopathy	48	15	-	-
Immunological disorder	5	2	-	-
PTGC	4	2	-	-
Morbus Castleman	2	-	-	-
Rheumatological disorder	2	-	-	-
Ruptured branchiogenic cyst	3	-	-	-
Venous malformation	1	1	-	-
Lymphangioma	1	-	-	-
Dermoid cysts	1	-	-	-
Isolated lymphadenopathy (%) *	21 (31.3)	6 (30.0)	0.91	-
Reactive/infective lymphadenopathy	13	5	-	-
PTGC	1	-	-	-
Morbus Castleman	1	-	-	-
Ruptured branchiogenic cyst	3	-	-	-
Venous malformation	1	1	-	-
Lymphangioma	1	-	-	-
Dermoid cysts	1	-	-	-

Table 1 describes the baseline characteristics of the malignant and benign group separately. Furthermore, differences between the different groups were investigated We calculated whether the input variable distribution was significantly different between the study and test cohort, for the whole set combined, and for the malignant and benign cases separately. Abbreviations: NA—not applicable; cHL—classical Hodgkin lymphoma; NLPHL—nodular lymphocyte-predominant Hodgkin lymphoma; ALCL—anaplastic large cell lymphoma; PMBCL—primary mediastinal large B-cell lymphoma; DLBCL—diffuse large B-cell lymphoma; BL—Burkitt lymphoma; T-LBL—T-cell lymphoblastic lymphoma; B-LBL—B-cell lymphoblastic lymphoma; PTGC—progressive transformation of germinal centers. * Isolated lymphadenopathy is defined as only one or two lymph nodes involved.

**Table 2:** Univariate analysis of all variables analyzed as prediction factors for malignancy.

Variables	Malignant Group (*n* = 115)			Benign Group (*n* = 67)			Total (*n* = 182)		Outcome Univariate Analysis		
	*n*	(%)	m	*n*	(%)	m	*n*	(%)	*p* Value	OR	95% CI
**Age**											
0–5 years	10	9	0	22	33	0	32	18	<0.001		
6–12 years	37	32		15	22		52	29			
13–18 years	68	59		30	45		98	55			
**Gender**											
Male	67	58	0	33	49	0	100	55	0.28	1.44	(0.79–2.64)
Female	48	42		34	51		82	45			
**Lymph nodes ultrasound**											
Pathological lymph nodes ^a^	101	96	10	24	37	2	125	74	**<0.001**	**43.14**	**(14.09–132.07)**
No/uncertain pathological lymph nodes	4	4		41	63		45	27			
**Cervical levels involved in accordance with Robbins Classification [49]**											
Level I	15	14	11	11	18	6	26	16	0.51	0.75	(0.32–1.76)
Level II	60	57		46	75		106	64	0.02	0.43	(0.21–0.86)
Level III	62	58		23	38		85	51	0.01	2.33	(1.22–4.44)
Level IV	91	86		18	30		109	65	<0.001	14.49	(6.67–31.47)
Level V	68	64		15	25		83	50	**<0.001**	**5.5**	**(2.71–11.11)**
Level VI	24	23		3	5		27	16	0.002	5.66	(1.63–19.68)
**Number of involved cervical levels**											
>3 levels involved	36	32	11	4	6	6	40	22	**<0.001**	**7.27**	**(2.46–21.51)**
**Cervical involvement**											
Unilateral	36	34	11	43	67	3	79	47	<0.001	3.98	(2.06–7.69)
Bilateral	70	66		21	33		91	54			
**Size lymph nodes level I, II, III (short axis)**											
>15 mm	22	77	0	14	42	0	36	56	0.03	3.31	(1.18–9.37)
**Size lymph nodes level IV, V, VI (short axis)**											
>10 mm	44	92	1	6	60	1	58	86	0.024	7.33	(1.44–37.22)
**Size non-cervical lymph nodes (short axis)**											
>10 mm	10	83	2	2	50	0	12	75	0.52	5.00	(0.42–59.68)
**Thorax abnormalities**											
Enlarged mediastinum	64	56	0	2	3	4	66	37	**<0.001**	**38.28**	**(8.93–164.10)**
Obstructed airway	32	28		1	2		33	19	<0.001	23.90	(3.18–179.72)
Trachea deviation	22	19		1	2		23	13	<0.001	14.67	(1.93–111.63)
V. cava superior syndrome	20	17		0	0		20	11	<0.001	0	NA
**Enlarged liver and/or spleen**											
No abnormalities	71	62	1	47	90	15	118	71	**<0.001**	**5.69**	**(2.10–15.43)**
Abnormal	43	38		5	10		48	29			
**Body regions involved (presence of pathological lymph node or mass)**											
High cervical	108	94	0	64	96	0	172	95	0.75	0.72	(0.18–2.89)
Supraclavicular	97	84		19	28		116	64	**<0.001**	**13.61**	**(6.55–28.29)**
Infraclavicular	39	34		0	0		39	21	**<0.001**	**0**	**NA**
Axilla	39	34		8	12		47	26	0.001	3.79	(1.65–8.71)
Mediastinal	90	78		5	8		95	52	**<0.001**	**44.64**	**(16.21–122.96)**
Abdominal lymph nodes	40	35		9	13		49	27	0.002	3.44	(1.54–7.65)
Other locations	69	60		16	24		85	47	<0.001	4.78	(2.44–9.38)
**Number of body regions involved**											
>3 involved	78	68	0	8	12	0	86	47	**<0.001**	**15.55**	**(6.74–35.86)**
**Laboratory values**											
ESR > 16.5 mm/h ^b^	77	72	8	28	43	2	105	61	<0.001	3.39	(1.77–6.48)
Hb < 8.15 g/dL ^c^	82	71	0	39	58	0	121	67	0.08	1.78	(0.95–3.36)
Leukocytes > 8.35 (×10^3^/mm^3^) ^c^	73	64	0	31	46	0	104	57	0.03	2.02	(1.09–3.72)
Neutrophils > 6.0 (×10^3^/mm^3^) ^b^	58	51	1	9	14	1	67	37	**<0.001**	**6.56**	**(2.97–14.49)**
Lymphocytes < 2.6 (×10^3^/mm^3^) ^b^	87	81	7	33	52	1	120	70	<0.001	3.39	(1.96–7.71)
Monocytes > 0.62 (×10^3^/mm^3^) ^b^	74	68	6	27	44	5	101	59	<0.001	3.26	(1.70–6.26)
Thrombocytes > 307.5 (×10^3^/mm^3^) ^b^	87	77	1	32	49	1	119	66	<0.001	3.42	(1.79–6.54)
Uric acid > 0.225 mg/dL ^d^	75	75	15	33	58	10	108	69	0.03	2.18	(1.09–4.37)
LD > 260.0 U/L ^b, f^	19	79	7	29	47	5	48	56	0.01	3.68	(1.23–11.03)
CRP > 3.35 µg/mL ^e^	78	82	20	20	36	11	98	65	**<0.001**	**8.26**	**(3.87–17.62)**
TARC > 850.0 pg/mL ^g^	69	90	7	3	8	30	72	63	**<0.001**	**97.75**	**(24.37–392.06)**
**Presence of B-symptoms**											
Presence of ≥1 B-symptoms	41	36	1	19	31	5	60	34	0.51	1.27	(0.66–2.46)
Weight loss	17	15	3	11	19	8	28	16	0.66	0.78	(0.34–1.79)
Night sweats	28	26	5	10	17	8	38	23	0.25	1.67	(0.74–3.74)
Fever	18	16	3	11	17	2	29	16	1.00	0.94	(0.41–2.14)

Abbreviations: ESR—erythrocyte sedimentation rate; Hb—hemoglobin; LD—lactate dehydrogenase; CRP—C-reactive protein; TARC—thymus and activation regulated chemokine; NA—not applicable; m—missing. ^a^ Ultrasound characteristics of pathological lymph nodes are diffuse hypo-echogenicity, absence of fatty hilum, round shaped and/or abnormal cluster of lymph nodes, and a resistance index (RI) above 0.8 [21,24,50]. The size of the lymph node that is considered pathological is dependent on the locations of the lymph node. Cervical lymph nodes in level two are considered pathological when the shortest diameter is larger than 15 mm. Cervical lymph nodes in other levels are considered pathological when the shortest diameter is larger than 10 mm. For non-cervical regions, the shortest diameter of greater than 10 mm was considered pathological [10,51]. We registered the lymph node as pathological when it was described as pathological by the radiologist based on the characteristics above. When the lymph node was described as doubtful pathological, we scored it as negative. ^b^ Conversion factor 1, ^c^ conversion factor 0.6206, ^d^ conversion factor 0.059, ^e^ conversion factor 10. ^f^ LD results are based on univariate analysis of NHL versus the benign group, since we concluded that LD is only a marker for NHL. ^g^ TARC results are based on univariate analysis of cHL versus benign group, since we concluded that TARC is only a marker for cHL. Outcomes in bold represent the variables that were included in the multivariate analysis.

## Data Availability

The data presented in this study are available on request from the corresponding author. The data are not publicly available due to ethical considerations. The codes of the machine learning model are available on request from the corresponding author.

## Supplementary Materials

The following supporting information can be downloaded at: https://www.mdpi.com/article/10.3390/cancers15041178/s1, Text S1: Methods section, Text S2: Explanation of the weighing factor, Table S1: Overview of the search of the literature; Table S2: Variables included in the analysis; Table S3: Specification of different localizations that were scored separately; Table S4: outcomes of the different tested models; Table S5: Feature importance in percentages and the final weighing factor; Figure S1: ROC curves of the cross validation; Figure S2: ROC curves of the different tested models. References [8,10,21,24,31,32,33,34,35,36,37,38,46,47,48,49,50,51,52,54,55] are also cited in the supplementary materials.

## List of Abbreviations

ALCL	Anaplastic large cell lymphoma
AUC	Area under the curve
BL	Burkitt lymphoma
B-LBL	B-cell lymphoblastic lymphoma
cHL	Classical Hodgkin lymphoma
CI	Confidence interval
CRP	C-reactive protein
CV	Cross validation
DLBCL	Diffuse large B-cell lymphoma
ELISA	Enzyme-linked immunosorbent assay
ESR	Erythrocyte sedimentation rate
LD	Lactate dehydrogenase
NHL	Non-Hodgkin lymphoma
NLPHL	Nodular lymphocyte-predominant Hodgkin lymphoma
OR	Odds ratio
PMBCL	Primary mediastinal large B-cell lymphoma
PTGC	Progressive transformation of germinal centers
PTLD	Post-transplant lymphoproliferative disorders
ROC	Receiver operating characteristic
TARC	Thymus and activation regulated chemokine
T-LBL	T-cell lymphoblastic lymphoma
