# Supplementary material for: Machine Learning Logistic Regression Model for Early Decision Making in Referral of Children with Cervical Lymphadenopathy Suspected of Lymphoma
Source: Cancers (Basel). 2023 Feb 12;15(4):1178. doi: 10.3390/cancers15041178 (PMC9954739; doi:10.3390/cancers15041178)
Supplement: Supplementary file 1 [file cancers-15-01178-s001.zip › cancers-2218811-supplementary.pdf]

Supplementary Materials: Machine Learning Logistic Regression Model for Early Decision Making in Referral of Children with Cervical Lymphadenopathy Suspected of Lymphoma

Text S1. Methods Section

We tested out several different model types, including easily explainable versions Decision Trees and Logistic Regression, and the higher quality models Random Forests and Support Vector Machines.

Logistic Regression is a machine learning classification algorithm and the classification version of linear regression. It is used to predict the probability of a categorical dependent variable. In logistic regression, the dependent variable is usually a binary variable showing true or false, or in our case malignant or benign.

Decision trees split up the dataset by evaluating it on a path of conditions. These conditions are based on single features in the dataset and intend to split the data as much as possible in the available classes. The goal is, at the end of the tree, in the leaves that the data are split up in separate classes, causing each path in the tree to correspond to a specific classification.

The random forest algorithm is similar to the decision tree as it is a collection of multiple trees. It takes subsets of the data and attempts to create the best decision tree for any of those subsets.. The sum of the decisions made by the decision trees is used in the end as the final classification [54].

Support Vector Machines is a supervised learning approach that can be used for both for classification andor regression analysis. It is mainly suitable to explain high-dimensional feature space by simulating the problem as a multidimensional space. It then identifies the shorted distance between same-class data points as well as keeping different classes away from each other, ideally to split the classes with one hyperplane corresponding to a function [55].

Text S2. Explanation of the weighing factor

The final formula for predictions with a logistic regression is the following:

$$P = \frac{1}{1 + e^{(\beta_o + \Sigma \beta_f x_f)}}$$

With P being the chance of a positive result, β a coefficient, o being an offset, f being features in the model and x being the value for each feature.

One can rewrite this formula in a formula suitable in the hospital:

$$\sum \beta_f x_f = log\left(\frac{1}{P} - 1\right) - B_o = C$$

With C being a cut-off for certain chosen values of P.

The final feature coefficients and offset were multiplied by 100 and rounded to halves for ease in day-to-day use as weighing factors (Figure 2 and Table S5).

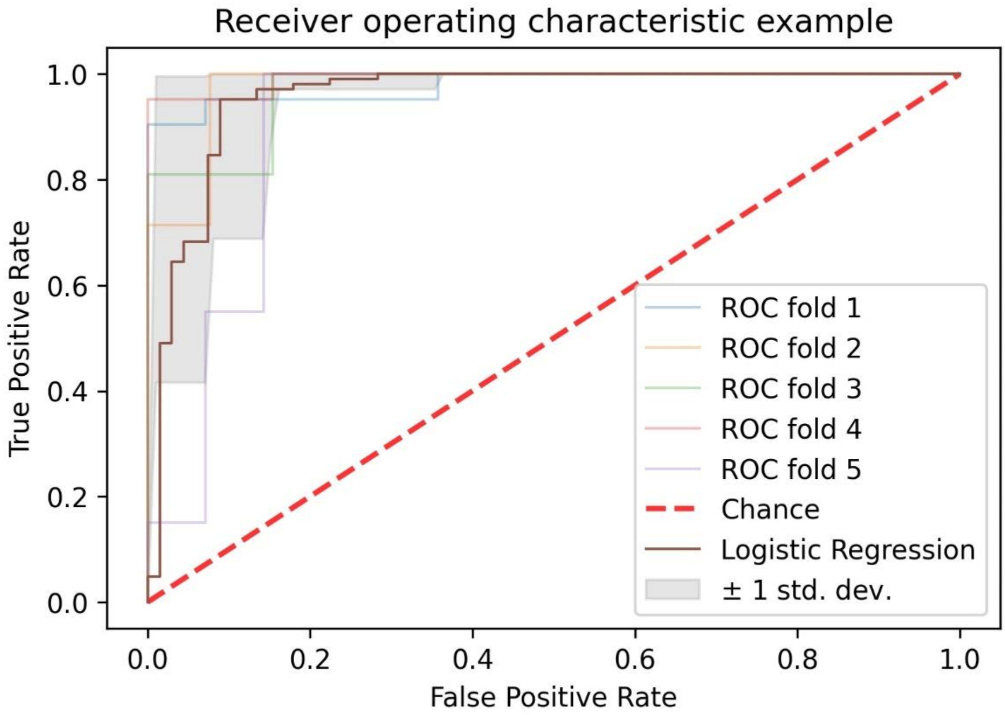

Figure S1. ROC curves of the 5-fold cross validation and the final Logistic Regression model.

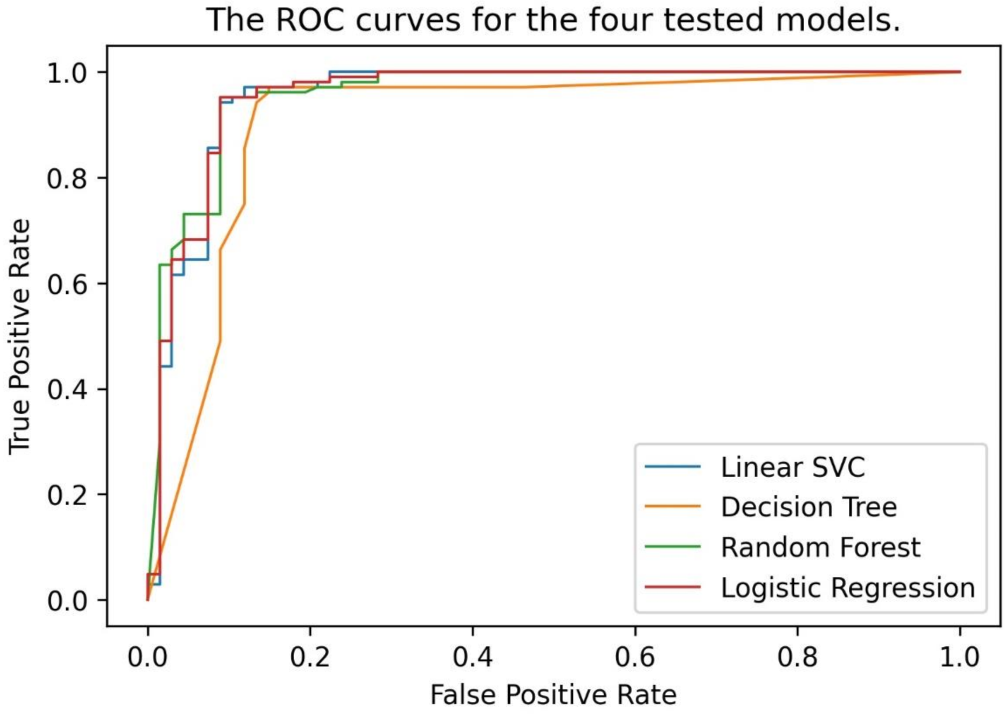

Figure S2. ROC curves of the different models that have been evaluated for our dataset.

Table S1. Overview of Our Literature Search.

Variable	Study	Year	Population	Outcome
Age	Celenk et al [34]	2015	Children, cervical lymphadenopathy	Older age associated with malignant disease, OR 1.07, p=0.046
	Sgro et al [35]	2021	Children, cervical lymphadenopathy	Older age associated with malignant disease, p < 0.01
	Karaman et al [31]	2010	Children, peripheral lymphadenopathy	Older age associated with malignant disease, p < 0.001

Gender	Celenk et al [34]	2015	Children, cervical lymphadenopathy	Male gender associated with malignant disease, Odd Ratio 4.184, p = 0.001
B-symptoms	Oguz et al [32]	2006	Children, peripheral lymphadenopathy	Associated with malignant disease, p=0.001
	Sgro et al [35]	2021	Children, cervical lymphadenopathy	Fever more often in benign diseases, p<0.001
ESR	Oguz et al [32]	2006	Children, peripheral lymphadenopathy	Elevated ESR associated with malignant disease, p=0.0001
CRP	Bozlak et al [36]	2016	Children, cervical lymphadenopathy	Elevated CRP associated with malignant disease, p = 0.001
	Oguz et al [32]	2006	Children, peripheral lymphadenopathy	Elevated CRP associated with malignant disease, p=0.0001
Hb	Bozlak et al [36]	2016	Children, cervical lymphadenopathy	Anemia associated with malignant disease, p=0.01
	Oguz et al [32]	2006	Children, peripheral lymphadenopathy	Anemia associated with malignant disease, p=0.0001
Leukocyte count	Oguz et al [32]	2006	Children, peripheral lymphadenopathy	Leukocytosis absent associated with malignant disease, p=0.017
Neutrophil count	Sgro et al [35]	2021	Children, cervical lymphadenopathy	Elevated neutrophils associated with malignant disease, p <0.001
Lymphocyte count	Sgro et al [35]	2021	Children, cervical lymphadenopathy	Children with benign diseases 2.5 ± 2.1 versus malignant diseases 2.1 ± 1, p=not applicable
Monocyte count	Sgro et al [35]	2021	Children, cervical lymphadenopathy	Children with benign diseases 0.53 ± 0.4 versus malignant diseases 0.76 ± 0.4, p=not applicable
Thrombocyte count	Sgro et al [35]	2021	Children, cervical lymphadenopathy	Children with benign diseases 294 ± 119 versus malignant diseases 355 ± 125, p=not applicable
LD	Venturini et al [8]	2020	Children, cervical lymphadenopathy	LD above 500 IU/mL was found in 25% of children with lymphoma/leukemia
	Sgro et al [35]	2021	Children, cervical lymphadenopathy	Higher LD associated with benign disease, p= 0.008
	Bozlak et al [36]	2016	Children, cervical lymphadenopathy	Higher LD associated with malignant disease, p = 0.034
	Oguz et al [32]	2006	Children, peripheral lymphadenopathy	Higher LD associated with malignant disease, p=0.001
Uric acid	Bozlak et al [36]	2016	Children, cervical lymphadenopathy	Higher uric acid levels common in malignant group, although not significant
TARC	Zijtregtop et al [38]	2021	Children, cHL versus controls	Elevated TARC associated with classical Hodgkin lymphoma, p < 0.01
Pathological lymph nodes US	Gupta et al [48]	2010	Different age groups, cervical lymphadenopathy	In malignant disease rounded shape, homogenous echotexture, peripheral vascularity, and significantly high resistance index
	Restrepo et al [51]	2009	Children, cervical lymphadenopathy	Round shape, absent or eccentric hilum, irregular borders, cystic necrosis, and chaotic capsular blood-flow pattern higher change of malignancy
Cervical levels	Wang et al [37]	2009	Children, cervical lymphadenopathy	More than one level associated with malignant disease; multivariate analysis OR 5.2, p=0,02
	Cunnane [47]		All ages, cervical lymphadenopathy	84.8% of patients with level V involvement had serious underlying pathology
Uni- of bilateral involvement	Srouji [52]	2004	Children, cervical lymphadenopathy	Bilateral lymphadenopathy most likely to be reactive
Size of the lymph node	Celenk [34]	2015	Children, cervical lymphadenopathy	Larger size associates with malignant disease, OR 1.445, p=0.038)
	Oguz et al [32]	2006	Children, peripheral lymphadenopathy	Size > 3 cm associated with malignant disease, p=0.02
	Sgro et al [35]	2021	Children, cervical lymphadenopathy	Malignant disease associated with larger size, p<0.001
X-thorax abnormalities	Soldes et al [33]	1999	Children, peripheral lymphadenopathy	Abnormalities associated with malignant disease, OR = 12.8, p < 0.01
	Sgro et al [35]	2021	Children, cervical lymphadenopathy	Abnormalities present in 37/49 of malignant cases (75.5%)
	Oguz et al [32]	2006	Children, peripheral lymphadenopathy	Abnormalities associated with malignant disease, p=0.001
Supraclavicular involvement	Soldes et al [33]	1999	Children, peripheral lymphadenopathy	Associated with malignant disease, OR = 10.9, p<0.01

	Oguz et al [32]	2006	Children, peripheral lymphadenopathy	Only present in malignant group
	Karaman et al [31]	2010	Children, peripheral lymphadenopathy	Associated with malignant disease, p < 0.05
Infraclavicular involvement	Bazemore et al [46]	2002	Alle ages, peripheral lymphadenopathy	Infraclavicular lymph nodes highly suspicious for malignancy
Axillary involvement	Karaman et al [31]	2010	Children, peripheral lymphadenopathy	Associated with malignant disease, p < 0.05
Mediastinal/hilar lymphadenopathy	Oguz et al [32]	2006	Children, peripheral lymphadenopathy	Associated with malignant disease, p=0.001
	Karaman et al [31]	2010	Children, peripheral lymphadenopathy	Associated with malignant disease, p < 0.04
Abdominal lymphadenopathy	Oguz et al [32]	2006	Children, peripheral lymphadenopathy	Associated with malignant disease, p=0.001
	Karaman et al [31]	2010	Children, peripheral lymphadenopathy	Associated with malignant disease, p < 0.03
Hepatosplenomegaly	Oguz et al [32]	2006	Children, peripheral lymphadenopathy	Associated with malignant disease, p=0.001
	Knight et al [50]	1982	Children, peripheral lymphadenopathy	Malignancy or other serious pathology was found in 50% of patients with hepatomegaly and 40% of patients with splenomegaly
More body regions involved/generalized disease	Oguz et al [32]	2006	Children, peripheral lymphadenopathy	Associated with malignant disease, p=0.02
	Soldes et al [33]	1999	Children, peripheral lymphadenopathy	More sites involved associated with lymphadenopathy, p< 0.05
	Karadeniz [49]	1999	Children, peripheral lymphadenopathy	Prevalence of malignancy with one lymph node group involved 1.4%, rising to 20% when four or more lymph node groups involved

Table S2. Variables included in the analysis.

**Patient Characteristics**	**Age,**<br>**Sex**
**Clinical signs**	The presence of B-symptoms in general<br>Unexplained fever > 38.5 C for at least 3 days<br>Drenching night sweats<br>Weight loss > 10% in 3 months
**Laboratory results**	ESR (mm/h)<br>Hb (g/dL)<br>Leukocyte count (x10^3/mm3)<br>Neutrophil count (x10^3/mm3)<br>Monocyte count (x10^3/mm3)<br>Lymphocyte count (x10^3/mm3)<br>Thrombocyte count (x10^3/mm3)<br>Uric acid (mg/dL)<br>LD (U/L)<br>CRP (µg/mL)<br>TARC (pg/mL)
**Imaging findings used for detection of the variables**	
Ultrasound neck	Pathological lymph nodes [a]<br>Unilateral or bilateral cervical lymph nodes<br>Involvement of cervical level I, II, III, IV, V and VI [b]<br>Total number of involved cervical levels<br>Size of largest lymph node per region: shortest and longest diameter
X-thorax	Enlarged mediastinum<br>Mediastinum/Hilar lymphadenopathy
X-thorax and/or CT thorax	Mediastinum/Hilar lymphadenopathy<br>Trachea deviation<br>Obstructed airway<br>Vena cava superior syndrome
Ultrasound abdomen	Hepatomegaly, splenomegaly or hepatosplenomegaly<br>Lymphadenopathy abdomen<br>Involvement organs
Other imaging modalities if available	Involvement of other body regions

[a]. Ultrasound characteristics of pathological lymph nodes are diffuse hypo-echogenicity, absence of fatty hilum, round shaped and/ or abnormal cluster of lymph nodes, and a Resistance Index (RI)

above 0.8 [21,24,50]. The size of the lymph node that is considered pathological is dependent on the localizations of the lymph node. Cervical lymph nodes in level two are considered pathological when the shortest diameter is larger than 15 mm. Cervical lymph nodes in other levels are considered pathological when the shortest diameter is larger than 10 mm. For non-cervical regions, the shortest diameter of greater than 10 mm was considered pathological [10,51]. We registered the lymph node as pathological when it was described as pathological by the radiologist based on the characteristics above. When the lymph node was described as doubtful pathological, we scored it as negative. [b]. We specified cervical lymphadenopathy using the Robbins' classification. Based on the Robbin's classification, the neck region is divided into six levels. Level I: submental and submandibular, level II: upper internal jugular, level III: mid internal jugular, level IV: lower internal jugular, level V: posterior triangle, level VI: anterior compartment. More than one level could be involved. Abbreviations: ESR Erythrocyte Sedimentation Rate; Hb Hemoglobin; LD Lactate Dehydrogenase; CRP C-reactive protein; TARC Thymus and activation regulated chemokine

Table S3. Specification of different localizations that were scored separately.

Nodal involvement	Upper cervical lymph nodes<br>Supraclavicular lymph nodes<br>Infraclavicular lymph nodes<br>Retro auricular or pre auricular lymph nodes<br>Waldeyer's ring<br>Mediastinum/hilar lymph nodes<br>Thoracic wall lymph nodes<br>Lymph nodes porta hepatis<br>Lymph nodes renal hilum<br>Lymph nodes splenic hilum<br>Other Abdominal lymph nodes (para-aortic, para-iliacal and mesenteric)<br>Inguinal lymph nodes
Extranodal involvement	Thyroid<br>Thymus<br>Lung<br>Liver<br>Spleen<br>Kidney<br>Intestines<br>Testis<br>Bone marrow<br>Bone<br>Other

The body region is involved when there is a pathological lymph node or mass detected. All the above-mentioned body regions were scored separately. Other body regions were scored as one point for each involved body region.

Table S4. Outcomes of the different model types with their precision scores.

**Model type**	**Model specifications**	**Sensitivity**	**Specificity**	**Likelihood ratio+**	**Likelihood ratio-**	**AUC**
Logistic Regression		95% (89% - 98%)	88% (77% - 94%)	7.97 (4.15-15)	0.05 (0.02-0.13)	92% (87%-96%)
Decision Tree	Function = Gini impurity<br>Max depth = 3	90% (83%-95%)	87% (76%-93%)	6.73 (3.65-12)	0.11 (0.06-0.20)	88% (83%-93%)
Random Forest	Function = Gini impurity<br>100 trees	94% (87%-98%)	91% (81%-96%)	11.0 (4.90-23)	0.06 (0.03-0.14)	93% (89%-97%)
Linear Support Vector Classifier	L2 penalty<br>Loss function = hinge<br>Tolerance = 0.0001<br>C = 1	95% (89%-98%)	90% (79%-95%)	12.0 (5.30-29)	0.07 (0.03-0.15)	92% (88%-97%)

Table S5. Feature importance in percentages and the final weighing factor.

Variable	Feature importance in percentage (%)	Weighing factor in the final model
Body regions > 3	15	15,5
Mediastinum/hilum involved	14	14,5
TARC	13	14
US pathological lymph nodes	11	11,5
Cervical levels > 3	8	9
Enlarged mediastinum	8	8,5
Supraclavicular lymph nodes	8	6,5
Cervical level V involved	6	6,5
Infraclavicular lymph nodes	6	6,0
Hepatosplenomegaly	5	5,0
Neutrophils	2	2,5
LD	2	2,5
